# Comparison of ESBL – And AmpC Producing *Enterobacteriaceae* and Methicillin-Resistant *Staphylococcus aureus* (MRSA) Isolated from Migratory and Resident Population of Rooks (*Corvus frugilegus*) in Austria

**DOI:** 10.1371/journal.pone.0084048

**Published:** 2013-12-31

**Authors:** Igor Loncaric, Gabrielle L. Stalder, Kemal Mehinagic, Renate Rosengarten, Franz Hoelzl, Felix Knauer, Chris Walzer

**Affiliations:** 1 Unit of Clinical Microbiology and Infection Biology, Institute of Bacteriology, Mycology and Hygiene, University of Veterinary Medicine Vienna, Austria; 2 Research Institute of Wildlife Ecology, Dept. of Integrative Biology and Evolution, University of Veterinary Medicine Vienna, Austria; Institut National de la Recherche Agronomique, France

## Abstract

In order to test whether rooks (*Corvus frugilegus*) represent good indicators for the potential circulation of antibiotics in their native habitat, two populations with different migratory behavior were tested for the presence of beta-lactamase producing *Enterobacteriaceae* and methicillin-resistant *Staphylococcus aureus* (MRSA). In all, 54 and 102 samples of fresh feces of a migratory and a resident population were investigated. A total of 24 and 3 cefotaxime-resistant enterobacterial isolates were obtained from the migratory and resident population, respectively. In these isolates CTX-M-1 (n = 15), CTX-M-3 (n = 3), and CTX-M-15 (n = 3) genes were detected. TEM-1 and OXA-1 were associated with CTX-M in 3 and 2 isolates, respectively. In two *E. coli* isolates CMY-2 could be detected, where from one isolate displayed an overexpression of chromosomal AmpC as well. Among *E. coli* isolates the most common phylogenetic group was A (n = 11) and ST1683 (n = 5). In one *E. coli* of B2-ST131 the *rfbO25b* locus was detected. Three *Enterobacter* isolates were stably derepressed AmpC-producers. In five samples of the migratory population, PVL positive MRSA could be isolated. Two isolates were typed SCC*mec* IVa, *spa* type t127, and ST1. Three isolates carried a SCC*mec* type IVc, with *spa* type t852 and ST22. The highly significant difference of the occurrence of antibiotic resistance between the migratory population from eastern Europe compared to resident population in our study indicates that rooks may be good indicator species for the evaluation of environmental contamination with antibiotic resistant bacteria, especially due to their ecology, foraging behavior and differing migratory behavior.

## Introduction

Antibiotic resistance in bacteria of animal origin and its zoonotic potential has elucidated considerable attention worldwide. Microorganisms responsible for increased resistance are often those from the group of gram-negative rods, mostly beta (β)-lactamase producing members of the family *Enterobacteriaceae*. Emergence and dissemination of resistance among *Enterobacteriaceae* represent a serious threat to public health [1]. Therefore, numerous studies involving these bacteria in humans and domestic animals have been published. However, there is still a scarcity of information on the presence of resistant *Enterobacteriaceae* isolates in wildlife. Studies on the presence of *Escherichia coli* (*E. coli*), especially extended-spectrum β-lactamase producing *E. coli* (ESBL) are performed to detect antibiotic resistance in populations of wild animals [2]. *E. coli* is a common inhabitant of the gastrointestinal tract of birds and mammals. It is also found in the environment and can therefore be used as indicator of antimicrobial resistance change [3]–[4]. To our knowledge there are no reports addressing isolation and characterization of β-lactamase producing *Enterobacteriaceae* from wildlife in Austria. Nevertheless, in the last years a few sources regarding wildlife resistant enterobacterial isolates from neighboring countries are reported. β-lactamase producing *E. coli* isolates were found in various mammalian and avian hosts in central Europe [5]–[12]. Studies addressing the presence of β-lactamase producing members of *Enterobacteriaceae* other than *E. coli* in wildlife are scarce [13]. Only few studies report the occurrence of another multi-drug resistant bacterial species: methicillin resistant *Staphylococcus aureus* (MRSA) in wildlife [14]–[15]. MRSA, first emerged in 1960s, is currently the most common multidrug-resistant bacteria causing nosocomial infections in Europe [16]. In the last years the transfer of MRSA isolates between animals and humans gained specific attention, especially in the case of MRSA multi locus sequence type (ST) 398 belonging to the clonal complex CC398, which has been the most commonly reported MRSA strain found in association with livestock (livestock-associated (LA)-MRSA) [17]. While studies on MRSA in humans, companion animals and livestock have been widely documented the role of this particular pathogen in wildlife is rarely studied. The emergence of MRSA in wild animals could represent a serious threat to human health, companion animals and livestock. Corvids and gulls feed on garbage in urbanized areas and are considered to be important reservoirs and vectors of resistant isolates in the environment [18]. Due to it's wide distribution and gregarious lifestyle the rook (*Corvus frugilegus*) is considered a good indicator for the potential circulation of antibiotics in their habitat. The biggest coherent population, comprising 5 to 10 million breeding pairs, is located in Russia [19]. Wintering grounds or resident populations range from the Czech Republic to France, mainly in the vicinity of cities. The objective of the study was to evaluate the occurrence of a) broad-spectrum-β-lactam resistance determinants among *Enterobacteriaceae* and b) MRSA isolated from feces of rooks (*Corvus frugilegus*) collected from a small resident Austrian population in Burgenland as well as an eastern European migratory population with wintering grounds in the city of Vienna.

## Methods

### Ethics Statement

No specific permission was required to non-invasively collect fresh fecal samples from the ground on public land. While the rook is protected in the EU birds directive (79/409/EWG) and in the respective national legislation in Austria this study did not involve a protected species. This field study did not involve any contact or disturbance of actual birds as fresh fecal samples were collected from the ground and tree leaves in public areas used by rooks on a daily basis. No permissions are required for this type of activity.

No animals were used, disturbed or otherwise impacted by this study and therefore no Institutional Animal Care and Use Committee (IACUC) permission was required.

#### Bacterial isolates

In March 2013, 54 (1 k–54 k) samples of fresh feces of a migratory population of rooks in the Lobau (lob, 48°10′59.78″N; 16°30′15.03″E) and 102 (55 k–106 k) samples of fresh feces of a resident, non-migratory population in Wulkaprodersdorf (wul, 47°47′18.30″N; 16°29′20.68″E) were collected in sterile tubes and transported directly to the laboratory. Each fecal sample was suspended in 1.5 ml of 0,9% sterile saline. For isolation of *Enterobacteriaceae*, 200 µl of suspended feces was precultured at 37°C overnight in buffered peptone water (BPW) (Merck, Germany) supplemented with cefotaxime (1 mg/L) and then cultivated at 37°C overnight on MacConkey agar (MCA) (Oxoid, Basingstoke, United Kingdom) supplemented with cefotaxime (1 mg/L), which select for broad-spectrum-cephalosporin-resistant isolates. For MRSA isolation, another 200 µl suspended feces was incubated at 37°C overnight in Trypticase soy broth (TSB) (Becton Dickinson, Heidelberg, Germany) with 6.5% NaCl and then streaked in a BBL™ CHROMagar™ MRSA II (Becton Dickinson, Heidelberg, Germany) and on Columbia CNA Agar with 5% Sheep Blood, Improved II (Becton Dickinson, Heidelberg, Germany) and incubated for at 35°C for 24 h.

After incubation on MCA one colony representing each a distinct colonial morphotype was regrown on the same plate and on Müller Hinton Agar II (Becton Dickinson, Heidelberg, Germany). For detection of members of *Enterobacteriaceae* standard bacteriological techniques were used. Isolates that were oxidase negative produced acid from glucose by oxidation as well as by fermentation were screened for ESBL production by combination disk tests using cefotaxime and ceftazidime with and without clavulanic acid (Becton Dickinson, Heidelberg, Germany) according to Clinical and Laboratory Standards Institute (CLSI) [20]. Furthermore, a cefoxitin disk (30 mg, Becton Dickinson, Heidelberg, Germany) was added to this test, to detect AmpC phenotypes. All isolates classified as intermediate or resistant using CLSI criteria (≤17 mm) to cefoxitin were suspected to be AmpC. ESBL as well as AmpC phenotype were confirmed by MASTDISCS™ ID AmpC and ESβL test (Mast Diagnostics, Merseyside, UK) and were subjected to further characterization. After incubation on BBL™ CHROMagar™ MRSA II the colonies showing typical colony appearance of MRSA were tested on production of β-lactamase using BBL™ DrySlide™ Nitrocefin (Becton Dickinson, Heidelberg, Germany).

### Identification of enterobacterial isolates

*E. coli*-like colonial morphotypes were tested using species specific PCR [21]. For species characterization of other enterobacterial isolates differ in colonial morhphology, 16S rDNA sequence analysis was performed [22]. The isolate was identified using the EzTaxon-e server (http://eztaxon-e.ezbiocloud.net/. Accessed 2013 September 15) [23]. All obtained sequences of have been deposited in the GenBank.

**Antimicrobial-susceptibility testing** of enterobacterial isolates was performed by the agar disk diffusion method according to CLSI for the following antibiotics: ampicillin, piperacillin, aztreonam, doripenem, ertapenem, imipenem, meropenem, gentamicin, tobramycin, amikacin, tetracyclin, ciprofloxacin, trimethoprim-sulfamethoxazole, chloramphenicol, fosfomycin (all from Becton Dickinson, Heidelberg, Germany). Additionally minimal inhibitory concentration (MIC) was performed by Etest (bioMérieux, Marcy l'Etoile, France) for cefoxitin (AmpC-hyperproducing isolates (stably derepressed only) and ceftazidime, and M.I.C. Evaluator test (Thermo Fisher, Basingstoke, UK) for ciprofloxacin, gentamicin, meropenem and cefotaxime. *Escherichia coli* ATCC 25922 was used as quality control strains for MIC tests. AmpC-hyperproducing isolates (stably derepressed) of *Enterobacter* sp. were defined as those with a cefotaxime and ceftazidfime MIC of ≥32 mg/L, without ESBL production and a negative cefoxitin-cefotaxime disk antagonist test [24]. MRSA susceptibility testing were performed as described elsewhere [22].

### Determination of genotypic resistance in enterobacterial isolates

Preliminary screening for the presence of *bla*_OXA_, *bla*_TEM_, *bla*_SHV_ and *bla*_CTX-M_ genes in the ESBL-positive isolates was performed by multiplex-PCRs as described earlier [25]–[26]. The gene that responded positively in the preliminary screening were further analysed by PCR and sequencing using primers as described elsewhere [27]–[28]. All isolates displaying AmpC phenotypes were tested to detect plasmid mediated AmpC β-lactamases (pAmpC) by multiplex PCR [29] which resulted in a positive amplicon corresponding to enzymes from the CIT group, which were further analyzed by PCR using CMY-F und CMY-R and sequenced as described elsewhere [27]. The sequences obtained were compared to those registered in the GenBank and compared with previously reported sequences for β-lactamases (http://www.lahey.org/studies/ - accessed August 2013). The *E. coli* isolates displaying AmpC phenotype were also tested for mutations in the chromosomal *ampC* promoter/attenuator region as described previously [30]. The presence of the genes non- β-lactamase genes *tet*(A), *tet*(B), *tet*(C), *tet*(D), *tet*(E), *tet*(G), *cat*, *cmlA*, *floR*, *sul1, sul2, sul3, strA, int1, int2*, variable regions of class 1 and class 2 integrons, and gene cassettes *dhfr1, dhfr12, dhfr17, aadA1, aadA2, aadA5, estX*, and *sat1* and *sat2* were tested with primers and under conditions as described elsewhere [31]. Isolates resistant to ciprofloxacin were tested for the plasmid-mediated quinolone restance genes, *qepA*, *qnrA*, *qnrB*, *qnrC*, *qnrD*, *qnrS*, and *aac(6′)-Ib-cr* by PCR [32]–[36].

### Analysis of genetic environment of blaCTX-M

The genetic environment of *bla*_CTX-M_ genes, such as upstream regions IS*Ecp1* and IS*26* was investigated by PCR and sequencing as previously described [31]. The IS*Ecp1* region was searched using IS*Ecp1* 5′ primer binding in the 5′ region of IS*Ecp1* and using ISE*cp1* UP primer binding to the transposase gene *tnpA* of IS*Ecp1*, located in the immediate blaCTX-M upstream region. The presence of IS*26* linked to *bla*_CTX-M_ was tested using *tnpA* IS*26* primer recognizing the transposase gene of the IS*26* element.

### Conjugations experiments

The transferability of *bla* genes was carried out by broth mating using a sodium azide-resistant *E. coli* J53 and sodium azide- and rifampicin resistant *E. coli* MT 102 as recipients. The strains were grown to the exponential phase and then mixed (1∶2), and the donor and recipient mixture was incubated in Müller-Hintorn (MH) broth overnight at 37°C. Transconjugants were selected on MH agar supplemented with sodium azide (200 mg/L), cefotaxime (1 mg/L), and MH supplemented with sodium azide (150 mg/L), rifampicin (50 mg/L), and cefotaxime (1 mg/L), respectively. Selected transconjugants were further characterized for their antimicrobial susceptibility, presence of the relevant *bla* genes, and co-transfer of other resistance genes as describe above for the wild isolates.

### Plasmid replicon typing

Plasmid DNAs were isolated using the GenElute™ Plasmid Miniprep Kit (Sigma-Aldrich, Vienna, Austria) according to the manufacturer's instructions. The presence of replicons was tested by PCR-based method as described previously [37].

### Molecular typing methods

The phylogenetic group of each *E. coli* isolate was determined by recently described revisited Clermont method [38]. Multilocus sequence typing (MLST) of *E. coli* isolates was performed as described previously [3]. Allelic profile and sequence type (ST) determinations were carried out according to the *E. coli* MLST website (http://mlst.ucc.ie/mlst/dbs/Ecoli. Accessed 2013 September 15.) scheme. In case of *E. coli* ST131, allele-specific PCR was performed to identify the O25 clone of *E. coli* [39]. Clonal diversity of enterobacterial isolates was assessed by enterobacterial repetitive intergenic consensus (ERIC)-PCR as described elsewhere [40].

### MRSA characterization

MRSA isolates were confirmed by *mecA* PCR and further investigated by the PCR [41] targeting Panton-Valentine leukocidin (PVL) genes, staphylococcal cassette chromosome *mec* (SCC*mec*) typing using multiplex PCR methods with subsequent subtyping, staphylococcal Protein A (*spa*) typing, and multiple-locus variable-number tandem repeat analyses (MLVA) using multiplex-PCR [41]. Detection of antibiotic resistance genes [42]–[43] and detection of pyrogenic exotoxin genes were as described earlier [44]. MLST was carried out by PCR amplification and sequencing of seven housekeeping genes (*arcC*, *aroE*, *glpF*, *gmk*, *pta*, *tpi*, *yqiL*) as previously described [45]. The allelic profiles and sequence types (ST) were assigned by the MLST web site (http://saureus.mlst.net/. Accessed 2013 September 15.).

### Statistical analysis

All statistical analyses were conducted using the statistical software R [46]. To test for differences of the frequencies of antibiotic resistances found in the Lobau population (lob) and the Wulkaprodasdorf population (wul) a poisson model (R function glm) was used (20 resistances). Since there was a clear difference in the frequencies between both populations, subsequently logistic regressions were conducted to post-hoc test every single antibiotic-resistance (156 samples each).

## Results

### Isolation of cefotaxime resistant isolates, phenotypic resistance in *Enterobacteriaceae* and species identification

Out of 54 samples, a total of 24 cefotaxime-resistant *Enterobactericeae* isolates were obtained from the migratory population, and 3 cefotaxime-resistant enterobacterial isolates were obtained from the resident population. 22 isolates displayed ESBL phenotype, and 5 isolates displayed AmpC phenotype, wherefrom three *Enterobacter* isolates were stably derepressed AmpC-producer. Apart from resistance to amipicillin and piperacillin antibiotics, resistance to tetracycline (85% of the isolates), trimethoprim-sulfamethoxazole (62%) and chloramphenicol (48%) was the most prevalent among isolates obtained from migratory population (Table 1). Three cefotaxime-resistant isolates retrieved from resident population showed phenotypic resistance to amipicillin and piperacillin (58 k, 69 k and 100 k) and tobramycin (isolate 58 k). All of enterobacterial isolates remained susceptible to carbapenems. The MICs of cefotaxime for all isolates ranged from 4 to >256 mg/L, the MIC of ceftazidime from 0.25 to 128 mg/L, the MIC for meropenem from 0.008 to 0.12 mg/L, the MIC for ciprofloxacin from 0.008 to >32 mg/L, and for gentamicin 0.5 and 64 mg/L (Table 1). Using species specific primers 19 isolates from migratory population as well as three from resident population were confirmed as *E. coli*. The highest 16S rDNA gene sequence similarity observed for the isolate 4.2 k (KF022262) was 99.65% with the type strain of *Klebsiella pneumoniae* subsp. *ozaenae* ATCC 11296^T^ (Y17654). Isolates isolate 24 k (KF022261) (99.5%) and 44.1 k (KF022259) (99.1%) showed highest similarity scores with type strain of *Enterobacter hormaechei* ATCC 49162^T^ (AFHR01000079). Isolate 22.1 k (KF022260) was most closely related with members of *Citrobacter youngae* group (*Citrobacter youngae* ATCC 29935^T^ (M59291)).

**Table 1:** Characterization of Extended-Spectrum-β-Lactamase-Producing, Plasmid-Mediated AmpC β-Lactamase-Producing and derepressed AmpC *Enterobacteriaceae* isolated from rooks.

					Upstream^c^ of *bla*_CTX-M_							MIC (mg/L)^h^					
Isolate	Species	*bla* gene	PG^a^	ST^b^	ISEcp1 5	ISEcp1 UP	*tnpA* IS26	Replicon^d^	Integron^e^	Non-β-lactam resistance pattern^f^	Non-β-lactam resistance genes^g^	CTX	CAZ	MEM	CIP	GEN	FOX
**2 k**	*E. coli*	CTX-M-1	B1	ST162	1,7	0,25	0,6	I1-Iy(+)	−	SXT(+), TET,	*tet*(A)*(+), sul1(+), sul2(+)*	16	1	0,008	0,015	0,5	n.a.
**7 k**	*E. coli*	CTX-M-1	A	ST744	−	0,25	0,6	N(+)	−	CHL(+), CIP(+), SXT(+), TET(+)	*tet*(B)*(+), cat(+), sul2(+), strA(+)*	32	2	0,008	8	0,5	n.a.
**8 k**	*E. coli*	TEM-15	A	ST23	n.a.	n.a.	n.a.	F(+), FIB(+), I1-Iy(+)	I1a	TET(+)	*sul2*	4	3	0,008	0,008	0,5	n.a.
**19 k**	*E. coli*	TEM-1, CTX-M-1	B1	ST58	−	0,25	0,6	F(+), FIB(+), I1-Iy(+), N(+), P	−	CIP, SXT(+), TET	*sul2(+), strA(+)*	16	1	0,008	4	0,5	n.a.
**23,2 k**	*E. coli*	TEM-1, CTX-M-1	B1	ST58	−	0,25	0,6	F(+), FIB(+), I1-Iy(+), N(+), P	−	CIP, SXT(+), TET	*sul2(+), strA(+)*	16	1	0,008	4	0,5	n.a.
**25 k**	*E. coli*	CTX-M-1	A	ST34	−	0,25	0,6	F(+), FIB(+), N(+)	−	CHL, GEN, SXT(+), TET	*tet*(A), *tet*(B)*(+), cat(+), strA(+)*	64	0,75	0,008	0,008	32	n.a.
**28 k**	*E. coli*	CTX-M-1	A	ST1683	−	0,25	0,6	HI1(+)	I1b	CHL(+), GEN(+), SXT(+), TET(+)	*tet*(A), *tet*(B)*(+), cat(+), sul1(+), sul2(+), strA(+)*	8	0,25	0,008	0,008	64	n.a.
**31,2 k**	*E. coli*	CTX-M-1	A	ST1683	−	0,25	0,6	F, HI1, N(+)	I1b	CHL(+), GEN, SXT(+), TET(+)	*tet*(B)*(+), cat(+), sul1, sul2(+), strA(+)*	16	1,5	0,008	0,015	64	n.a.
**32 k**	*E. coli*	CTX-M-15	B2	ST131	−	0,2	0,9	F(+), FIA(+), FIB(+), Y	I1b	CIP(+), SXT(+), TOB(+)	*sul1(+), strA(+)*	>256	6	0,015	>32	1	n.a.
**33 k**	*E. coli*	CMY-2	E	NT	n.a.	n.a.	n.a.	NT	−	−	*−*	8	8	0,015	0,015	0,5	n.a.
**38 k**	*E. coli*	OXA-1, CTX-M-15	A	ST90	−	0,2	0,9	F(+), FIA(+), FIB(+)	I1b	CIP(+), SXT(+), TET(+), TOB(+)	*tet*(A)*(+), sul1(+)*	64	12	0,008	>32	1	n.a.
**39 k**	*E. coli*	CTX-M-1	A	ST34	−	0,25	0,6	F(+), FIB(+), N(+)	−	CHL, GEN, SXT(+), TET	*tet*(A), *tet*(B)*(+), cat(+), strA(+)*	64	0,75	0,008	0,008	32	n.a.
**43,3 k**	*E. coli*	CTX-M-1	A	ST1683	−	0,25	0,6	HI1(+)	I1c	CHL(+), GEN(+), SXT(+), TET(+)	*tet*(B)*(+), cat(+), sul2(+), strA*	8	0,38	0,008	0,008	32	n.a.
**45,1 k**	*E. coli*	CTX-M-1	A	ST1683	−	0,25	0,6	HI1	I1b	CHL(+), GEN(+), SXT(+), TET(+)	*tet*(A)*(+), tet*(B)*(+), cat(+), sul1(+), sul2(+), strA(+)*	16	0,25	0,008	0,015	64	n.a.
**46,2 k**	*E. coli*	CTX-M-3	A	ST744	−	−	−	F(+)	−	AMK, CIP(+), CHL, FOF(+), GEN, SXT, TET(+)	*tet*(B)*(+), sul2, strA(+)*	8	1	0,008	8	0,5	n.a.
**47,1 k**	*E. coli*	CTX-M-1	C	ST744	−	0,25	0,6	N(+)	−	CIP, TET	*tet*(B)*(+), cat(+), sul2, strA(+)*	16	2	0,008	16	0,5	n.a.
**47,2 k**	*E. coli*	CMY-2	B1	ST224	n.a.	n.a.	n.a.	FIA, I1-Iy	−	CHL, CIP, SXT, TET	*tet*(A), *floR, sul2, strA(+)*	8	24	0,008	>32	1	n.a.
**48,1 k**	*E. coli*	CTX-M-1	B1	ST162	−	0,25	0,6	NT	−	TET	*tet*(A)	32	1	0,015	0,25	1	n.a.
**53 k**	*E. coli*	CTX-M-1	A	ST1683	−	0,25	0,6	HI1	I1b	CHL(+), GEN(+), SXT(+), TET(+)	*tet*(A)*(+), tet*(B)*(+), cat(+), sul1(+), sul2(+), strA(+)*	16	0,25	0,008	0,015	64	n.a.
**58 k**	*E. coli*	OXA-1, CTX-M-15	B2	ST491	−	0,2	0,9	F(+), FIA(+), FIB(+), HI1	I1b	CIP, SXT, TOB	*sul1*	64	12	0,008	0,12	0,5	n.a.
**69 k**	*E. coli*	CTX-M-1	D	ST69	1,7	0,25	−	F, FIB, I1-Iy(+)	−	−	−	32	1	0,015	0,015	1	n.a.
**100 k**	*E. coli*	CTX-M-1	D	ST69	1,7	0,25	−	F, FIB, I1-Iy(+)	−	−	−	32	1	0,015	0,015	1	n.a.
**4,2 k**	*Klebsiella* sp.	TEM-1, CTX-M-3	n.a.	n.a.	−	0,3	−	FIAs	−	CHL, TET	−	32	1,5	0,008	0,5	0,5	n.a.
**22,1 k**	*Citrobacter* sp.	CTX-M-3	n.a.	n.a.	1,7	0,3	−	NT	−	−	−	16	0,75	0,06	0,015	0,5	n.a.
**11,2 k**	*Enterobacter* sp.	dAmpC	n.a.	n.a.	n.a.	n.a.	n.a.	n.a.	n.a.	−	−	>256	96	0,12	0,015	0,5	>256
**24 k**	*Enterobacter* sp.	dAmpC	n.a.	n.a.	n.a.	n.a.	n.a.	n.a.	n.a.	−	−	>256	128	0,12	0,03	0,5	>256
**44,1 k**	*Enterobacter* sp.	dAmpC	n.a.	n.a.	n.a.	n.a.	n.a.	n.a.	n.a.	−	−	>256	96	0,12	0,015	0,5	>256

^a^ Phylogenetic group.

^b^ Sequence type.

^c^ ISEcp1 5′, nexus between the 5′ region of IS*Ecp1* and *bla*_CTX-M_; IS*Ecp1* UP, nexus between IS*Ecp1* and *bla*_CTX-M_ using forward primer for transposase gene of IS*Ecp1* and reverse primer for *bla*_CTX-M_; *tnpA* IS*26*, nexus between IS*26* and *bla*_CTX-M_.

^d^ in parenthesis (+) means positive after testing of transconjugants.

^e^ I1a, class 1 integron 1.7 kb: *dfr17-aadA5*; I1b, class 1 integron 1.7 kb: *dfr17*; I1c2, class 1 integron 1.75 kb: none of cassettes could be detected.

^f^ in parenthesis (+) means positive after testing of transconjugants. Abbreviations: AMK, amikacin; CHL, chloramphenicol; CIP, ciprofloxacin; FOF, fosfomycin; GEN, gentamicin; SXT, trimethoprim-sulfamethoxazole; TET, tetracycline; TOB, tobramycin.

^g^ in parenthesis (+) means positive after testing of transconjugants.

^h^ Abbreviations: CTX, cefotaxime; CAZ, ceftazidime; MEM, meropenem; CIP, ciprofloxacin; GEN, gentamicin; FOX, cefoxitin.

### Characterization of genotypic resistance in *Enterobacteriaceae*

The most prevalent genes in ESBL-producing isolates were those from the *bla*_CTX-M_ group. In all but one isolate from both rook populations *bla*_CTX-M_ was detected. The genes *bla*_CTX-M-1_, *bla*_CTX-M-3_, or *bla*_CTX-M-15_ were detected solely in 15, 2, and 1 isolates, respectively. The gene *bla*_CTX-M-1_ was detected in combination with *bla*_TEM-1_ in 2 isolates, *bla*_CTX-M-15_ was detected in combination with *bla*_OXA-1_ in one isolate from the resident and in one from the migratory population. The gene *bla*_CTX-M-3_ was detected in a *Citrobacter* isolate as well as in a *Klebsiella* isolate in combination with *bla*_TEM-1_. *bla*_TEM-15_ was detected in one *E. coli* isolate. In one *E. coli* (47.2 k) isolate displaying AmpC phenotyp the combination of plasmidic AmpC (CMY-2) and overexpression of chromosomal AmpC was detected. Mutations in the promoter and attenuator region of the chromosomal *ampC* gene from *E. coli* were found at positions −18 (G→A), −1 (C→T), and +58 (C→T) of the *ampC* gene of *E. coli* K12. In one *E. coli* (33 k) isolate CMY-2 was solely detected. The non-β-lactamase genes *strA* (n = 14), *sul2* (n = 13), *and tetB* (n = 10) were predominant (Table 1). Class 1 integron was detected in 9 ESBL-producing *E. coli* isolates. Seven isolates contained an integron, 1.7 kb in size with *dfr17*-*aadA5* cassettes. One isolate contained a 1.7 kb integron with *dfr17 cassete*, and in one isolate with a 1.7 kb integron none of the cassettes tested could be detected. Even though phenotypic resistance to ciprofloxacin was detected in seven isolates in the wild isolates as well as in some plasmids, none of the plasmid-mediated quinolone resistance genes were detected. Phenotypes and resistance genes, including integrons and gene cassettes, are summarized in Table 1.

### Conjugations experiments and plasmid analysis

The transfer of the ESBL and pAmpC producing isolates to sodium azide-resistant *E. coli* J53 and sodium azide- and rifampicin resistant *E. coli* MT 102 was demonstrated for all *bla* positive *E. coli* isolates. The *bla*_CTX-M-3_ of the *Citrobacter* isolate and *bla*_CTX-M-3_/*bla*_TEM-1_ of the *Klebsiella* isolate did not transfer. Resistance to non-β-lactamase genes was also cotransferred in some cases (Table 1). The replicon typing revealed 13 different replicon types. Replicon type F was the most common replicon among the isolates.

### Genetic environment of *bla*_CTX-M_

PCRs applied different primer combination identified the insertion sequence IS*Ecp1* in its entirety or partially truncated in 20 isolates (Table 1.). PCR using primer IS*Ecp1* 5′ indicated a ∼1.7 kb amplicon of the entire IS*Ecp1* in two isolates (2 k and 100 k). 16 isolates contained the transposase gene of the insertion sequence IS*26* as analyzed by PCR. As confirmed with sequencing all these isolates showed the presence of IS*26* disrupted by IS*Ecp1*. All CTX-M-1-positive isolates had a 48 bp region (W sequence) upstream of the *bla*_CTX-M-1_ gene and an additional 32 bp X sequence, as observed in a previous sequence reported in the GenBank database (accession no. AM003904). The CTX-M-15-positive isolates had only the 48 bp W sequence, as previously described (AM040707).

### Enterobacterial molecular typing methods

Eleven *E. coli* isolates belonged to phylogenetic group A, 4 to B1, 2 to B2, 1 to C, 2 to D and one to E. *E. coli* MLST typing identified 11 different types among the tested isolates (Table 1). Among *E. coli* isolates, the most commonly identified genotypes were ST1683 (n = 5), ST744 (n = 3), ST34 (n = 2), ST58 (n = 2), ST69 (n = 2), and ST162 (n = 2), whereas isolates belonging to the ST23, ST90, and ST131 genotypes were also identified. The CMY-2 isolate belonged to ST224. One CMY-2 isolate could not be assigned. Allele-specific PCR confirmed the presence of the *rfbO25b* locus in *E. coli* of ST131 (phylogenetic group B2) and, showing that it belonged to the O25b type. ERIC-PCR analysis was used to analyze the molecular epidemiology of all enterobacterial isolates and showed that two ST162 (B1) isolates (2 k and 48,1 k), two ST1683 (A) (45,1 k and 53 k), two ST34 (A) (25 k and 39 k), two ST58 (B1) (19 k and 23,2 k), two ST69 (D) isolates from resident population (69 k and 100 k) as well as two derepressed AmpC *Enterobacter* sp. were clonally related due to identical ERIC fingerprint (data not shown).

### MRSA characterization

In five samples of the migratory population, MRSA could be isolated, whereas all fecal samples of the resident population were negative on presence of MRSA. In all five isolates the production of β-lactamase was confirmed using a nitrocefin assay. All isolates were *mecA* and PVL positive as determined by PCR. Two isolates (3mrsak and 5mrsak) carried a SCC*mec* type IVa cassette, belonged to *spa* type t127, and shared the same MLVA genotype. These two isolates carried *tet*(K) resistance genes as well as *seh* toxin genes. MLST revealed that these two isolates belonged to sequence type (ST) 1. Other three isolates (39mrsak, 41mrsak, 53mrsak) carried a SCC*mec* type IVc, with *spa* type t852 and were indistinguishable after multiplex-PCR MLVA, carried *aacA-aphD* and *msrA* resistance genes and *sed* toxin genes. These isolates were typed by MLST as ST22. Isolates 3mrsak and 5mrsak were resistant to β-lactams and tetracycline, whereas 39mrsak, 41mrsak and 53mrsak were resistant to β-lactams and ciprofloxacin. The MICs of oxacillin for all isolates ranged from 32 to >256 mg/L, the MIC of cefoxitin from 24 to 48 mg/L (Table 2).

**Table 2:** Characteristics of Methicillin-resistant *Staphylococcus aureus* (MRSA) isolated from rooks.

					MIC (mg/L)^c^			
Isolate	PVL^a^	SCC*mec* type	*spa* type	ST^b^	FOX, OX	Resistance phenotype^d^	Resistance genotype	Exotoxin genes
3mrsak	+	IVa	t127	ST1	48, >256	β-lactams, TET, CIP	*tet*(K)	*seh*
5mrsak	+	IVa	t127	ST1	48, >256	β-lactams, TET, CIP	*tet*(K)	*seh*
39mrsak	+	IVc	t852	ST22	24, 32	β-lactams, GEN, CIP	*aacA-aphD, msr*(A)	*sed*
41mrsak	+	IVc	t852	ST22	24, 32	β-lactams, GEN, CIP	*aacA-aphD, msr*(A)	*sed*
53mrsak	+	IVc	t852	ST22	24, 32	β-lactams, GEN, CIP	*aacA-aphD, msr*(A)	*sed*

^a^ Panton-Valentine leukocidin.

^b^ Sequence type.

^c^ OX, oxacillin; FOX, cefoxitin.

^d^ Antibiotics tested: penicillin, oxacillin, cefoxitin, cefovecin, cefquinome, tetracycline (TET), ciprofloxacin (CIP), gentamicin (GEN), chloramphenicol, erythromycin, clindamycin, teicoplanin, trimethoprim/sulfamethoxazole, linezolid, rifampicin and mupirocin.

### Differences in enterobacterial antibiotic resistance between the two rook populations

**Figure 1:**
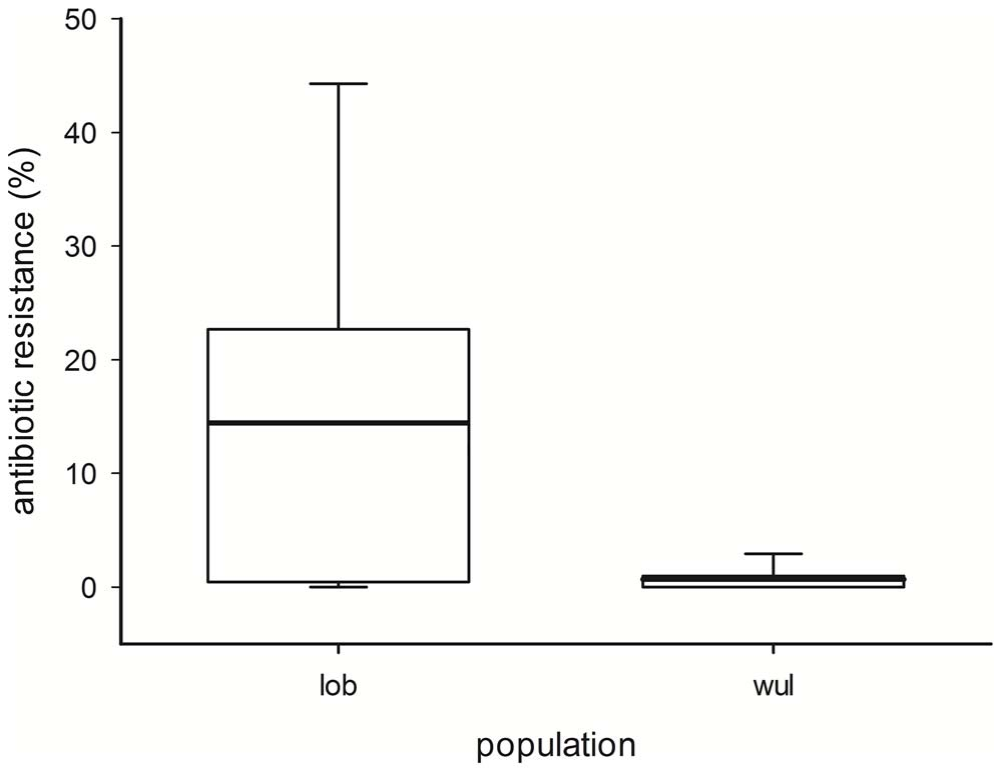
Proportion of antibiotic resistance in the two rook populations. The percentage of antibiotic resistance rates is significantly (p<0.001) higher in the migrating (lob) population then in the resident (wul) population

A total of 44.4% of the fecal samples (24 of 54) collected in the migrating population showed at least resistance to one of the tested antibiotics compared to 2.9% (3 of 102) in the resident population. As shown in Figure 1 the proportion of antibiotic resistances is significantly (p<0.001) higher in the migrating (lob) population then in the resident (wul) population. Posthoc tests for each tested antibiotic showed significant differences for ampicillin, piperacillin, ceftazidime, cefotaxime, aztreonam and trimethoprim-sulfamethoxazole (Table 3).

**Table 3:** Differences in the occurrence of antibiotic resistance between the migratory population (lob) and a the resistant population (wul) of rooks.

Tested antibiotic	Migratory population (n = 54)		Resident population (n = 102)		p-value
Ampicillin	24	(44.44)	3	(2.94)	≤0.001
Piperacillin	23	(42.59)	3	(2.94)	≤0.001
Ceftazidime	7	(12.96)	1	(0.98)	≤0.050
Cefotaxime	24	(44.44)	3	(2.94)	≤0.001
Cefepim	0	(0.00)	0	(0.00)	ns
Doripenem	0	(0.00)	0	(0.00)	ns
Imipenem	0	(0.00)	0	(0.00)	ns
Meropenem	0	(0.00)	0	(0.00)	ns
Ertapenem	0	(0.00)	0	(0.00)	ns
Aztreonam	6	(11.11)	1	(0.98)	≤0.050
Ciprofloxacin	8	(14.81)	0	(0.00)	ns
Amikacin	2	(3.70)	0	(0.00)	ns
Gentamicin	7	(12.96)	0	(0.00)	ns
Tobramycin	1	(1.85)	1	(0.98)	ns
Tetracyclin	18	(33.33)	0	(0.00)	ns
Chloramphenicol	10	(18.52)	0	(0.00)	ns
Trimethoprim-Sulfamethoxazole	13	(24.07)	1	(0.98)	≤0.005
Fosfomycin	1	(1.85)	0	(0.00)	ns

The number of resistant individuals per group respectively the percentage of resistant individuals per study site (in brackets) differs significantly between in the two populations for ampicillin, piperacillin, ceftazidime, cefotaxime, aztreonam and trimethoprim-sulfamethoxazole (ns indicates a non significant difference).

## Discussion

In the present study, the phenotypic and genotypic characteristics of β-lactamase producing *Enterobacteriaceae* as well as methicillin resistant *Staphylococcus aureus* isolates from two different rooks populations were investigated. The isolation rates (44.4%) of cefotaxime resistant enterobacterial isolates was significantly higher in migratory population (2,9%) than in the resident population.

Genotypic characterization of the isolates revealed that the majority of ESBL-*E. coli* harbored *bla*_CTX-M-1_ genes and belonged to six different STs: ST34 (phylogenetic group A), ST58 (B1), ST69 (D), ST162 (B1), ST744 (A and C), and ST1683 (A). CTX-M-1, a major ESBL type in livestock in Europe [47] has also been detected in different wildlife avian host in Europe such as different birds of prey from Portugal, seagulls from Portugal and Sweden, black headed gull from Czech Republic and yellow legged gull from France [6] [48]–[51]. Very recently the *E. coli* harboring *bla*_CTX-M-1_ and *bla*_TEM-1_ belonged to ST744 (A) were isolated from different birds of prey (*Milvus migrans*, *M. milvus* and *Buteo buteo*) in Germany [52]. In our study two *E. coli* CTX-M-1 clonally related isolates also harbored *bla*_TEM-1_ and belonged to ST58. ST58 isolates harboring the same genes were also isolated from seagulls in Portugal [53]. Two CTX-M-1 *E. coli* isolates retrieved from the resident rook's population belonged to the phylogenetic group D and ST69. This is especially interesting because previous studies have reported the association of *E. coli* isolates of group D with extra-intestinal infections in humans [54]. ST69 has been also identified among CTX positive *E. coli* isolates from seagulls [53]. Three isolates, one *E. coli* and two isolates, identified based on their 16S rDNA similarity as a members of genera *Citrobacter* and *Klebisiella* from migratory population carried *bla*_CTX-M-3_ genes, The *Klebsiella* sp. isolate harbored also the *bla*_TEM-1_ gene. To the authors knowledge, there are no reports describing isolation of *E. coli, Citrobacter* sp. and/or *Klebsiella* sp. harboring CTX-M-3 type alone or in combinations with other β-lactamases from rooks. Two *E. coli* (32 k and 38 k) isolates from the migratory as well as one (58 k) from the resident population harbored the *bla*_CTX-M-15_ gene, which is the dominant ESBL gene in strains isolated from humans and is rarely found in animals [55]–[57]. CTX-M-15 isolates from this study belonged to ST90 (A), ST491 (B2) and ST131 (B2), wherefrom isolates 38 k and 58 k carried a *bla*_OXA-1_ gene as well. CTX-M-15 producing ST90 (A) *E. coli* variant has been detected previously from yellow legged gulls in the South of France [51], whereas ST491 (B2) has not yet been detected among wildlife. In one fecal sample of the migratory population, the currently spreading pandemic CTX-M-15-producing *E. coli* clone B2-O25b-ST131population was isolated. The B2-O25b-ST131 clone presents an anthropogenic zoonotic pathogen [58] with high extraintestinal virulence and is described to cause urinary infections, bacteraemia, and sepsis [58]–[59]. Pandemic spread of B2-O25b-ST131 has also been reflected in wildlife. This clone was isolated not only from wildlife in urban areas, as detected in *E. coli* harbouring the CTX-M-9 type in a feral urban brown rat (*Rattus norvegicus*) [60], but also in Glaucous-winged gull on the remote Commander Islands in Russia [61]. Association of IS*26* and *bla*_CTX-M-15_ in isolate 32 k has been observed previously in the B2-O25b-ST131 clone [31] [62]. One cefotaxime-resistant *E. coli* isolate (8 k) harboring the *bla*_TEM-15_ gene belonged to ST23 (A). This type of *E. coli* has not been detected yet in rooks.

During this study three kind of AmpC-producing *Enterobacteriaceae* were isolated, plasmid mediated AmpC (pAmpC) genes in *E. coli* isolates, combination of pAmpC and overexpression of chromosomal AmpC, and derepressed AmpC in three *Enterobacter* sp. isolates. The presence of pAmpC β-lactamases have been found worldwide but are less common than ESBLs [63]. Furthermore two *E. coli* isolates harbored pAmpC. Among the AmpC β-lactamases, *bla*_CMY-2_ is by far the most common worldwide, which was detected in isolates from human, livestock and companion animals [47] [63]. The information on the presence of AmpC producing *Enterobacteriacea* in wildlife is scarce. Recently, *E. coli* harboring CMY-2 belonged to different phylogenetic groups and STs were isolated from wild coastline birds in Miami Beach, Florida, USA [13]. Among others, one *E. coli* isolate belonged to B1-ST224. *E. coli* isolate 47.2 k analyzed in this study belonged also to the same phylogenetic group and shared the same ST with those from USA. Based on the results obtained, three *Enterobacter* isolates most closely related to type strain of *Enterobacter hormaechei*, were detected as a derepressed AmpC mutants. Derepressed production of AmpC β-lactamase has been documented to be a predominant mechanism of extended-spectrum cephalosporin resistance in clinical isolates of *Enterobacter* spp. [64]–[66]. To the authors' knowledge there are no current reports describing the isolation of stably derepressed AmpC-producing *Enterobacter* isolates from wildlife. In the present study, for all *E. coli* isolates, the *bla* genes could be transferred by conjugation to both *E. coli* recipient strains. Additionally, the majority of non-β-lactamase genes were co-transferred. Our results indicate that the horizontal dissemination of *bla* genes is due to multiple plasmids, because the majority of replicon types were detected both on conjugative plasmids as well as in the wild isolates. Association of IS*Ecp1* and *bla*_CTX-M_ was observed in all but one *bla*_CTX-M_ isolates. The presence of IS*Ecp1* element upstream of *bla*_CTX-M_ in *E. coli* isolates originated from wildlife has been observed previously [12]. Genotyping of the enterobacterial isolates by ERIC-PCR showed that some are genetically homogeneous. These isolates have all other characteristics in common (*bla* genes, in case of *E. coli* belonged to the same phylogenetic group and ST type, replicon combination, resistance pattern) indicating that resistances to antibiotics spread, not only due to dissemination of different β-lactamases, but also due to clonal transmission. This is probable, as they originated from rooks sharing the same habitat.

Together with the isolation of pandemic CTX-M-15-producing *E. coli* clone B2-O25b-ST131, the isolation of MRSA isolates, even in a low prevalence (9,4% in migratory population and none in the resident), is an important finding as there are only few reports regarding this issue. In 2012, three MRSA isolates from lesser yellowlegs (*Tringa flavipes*) and eastern cottontails (*Sylvilagus floridanus*) in Central Iowa, USA have been isolated [14]. These isolates were *spa* typed as t002 and t008, wherefrom t008 was PVL positive. MRSA was also detected in brown rats (*Rattus norvegicus*), chaffinch (*Fringilla coelebs*), common seals (*Phoca vitulina*) [67], and from a hedgehog [68] but isolates from these wild animals were MRSAs carrying the novel *mecC* gene. Recently, our working group also detected *mecC* positive MRSA isolates in wildlife, particularly in European brown hare (*Lepus europaeus*), European otter (*Lutra lutra*) and a European hedgehog (*Erinaceus europaeus*) [22]. Very recently, 12 out of 1342 tested wild animals from Spain (Red deer, Iberian ibex, Wild boar, Eurasian Griffon vulture) were tested MRSA positive. Among them, two isolates belonging to t127, ST1 were recovered from two wild boars [15]. MRSA isolates 3mrsak and 5mrsak MRSAs had all characteristics in common, carried SCC*mec* IVa, belonged to *spa* type t127 and ST1, were PVL positive. MRSAs belonging to SCC*mec* IVa, t127, ST1 are commonly isolated from humans as well as from animals [69]. From Austria, a MRSA with SCC*mec* IVa, t127, ST1 has been reported. Three SCC*mec* IVa, t127, ST1 MRSA isolates originated from a veterinary hospital, (University of Veterinary Medicine, Vienna) and caused infections in horses. Two isolates originated from horse-associated personnel. All five isolates were PVL negative [70]. Unlike reports from other European countries [69] [71] there are no studies describing the isolation of MRSA belonging to t127, ST1 from livestock in Austria. The other three PVL positive MRSA isolated during this study belonged to *spa* type t852 and ST22 and carried SCC*mec* type IVc are a variant of epidemic methicillin-resistant *Staphylococcus aureus* (EMRSA-15) [72]. EMRSA-15 variants occur worldwide and were isolated from humans as well as from animals [73]–[74]. In Europe, t852, ST 22 PVL-MRSA strains are mainly reported from North-West Europe [72] [75]–[76]. Interestingly, there are no reports on presence of t852 MRSA in Austria, and no entries in the Ridom spaserver database (http://spaserver.ridom.de/, Accessed 2013 August 26.). To the authors' knowledge there are no reports on presence of these MRSA variants in rooks.

Based on the significant differences recorded between the migratory and non-migratory populations, it is the authors' opinion that rooks may be good indicator species for the evaluation of environmental contamination with antibiotic resistant bacteria. Populations originating from North and Central-Russia to the Ural, travel the longest distance by migrating along the Baltic to Western Europe (Ukraine, Poland, Czech Republic, Slovakia over Germany, South- West France until North-England). Rook-flocks wintering in Vienna mostly originate from Russia, Belarus, Poland and the region North-East of the Carpathian mountains and arrive regularly every winter with the first small flocks of rooks usually being observed in the beginning of October followed by a peak in the middle of the month [19].

Insufficient degradation of antibiotics in wastewater treatment plants results in antibiotic residues entering the environment in moderate concentrations, and over time, selection pressure enhances survival of organisms bearing resistance traits [77]–[78]. The spread of manure containing resistant organisms onto crop fields is another path of how antibiotics or resistant bacteria can enter the environment [52]. The highly significant difference in the occurrence of antibiotic resistance between the migratory population from eastern Europe compared to the non-migratory, resident population in our study indicates that rooks may provide a sufficiently good resolution for the evaluation of environmental contamination with antibiotic resistant bacteria, especially due to their ecology, foraging behavior and differing migratory behavior.

In conclusion, our study contributes to the growing evidence that multidrug resistant members of *Enterobacteriaceae* as well as MRSA can be found in wildlife. The presence of pandemic isolates in wildlife emphasizes the complexity in the dissemination of antimicrobial drug resistance. Moreover, the present study confirmed that rooks might represent an important vector not only of β-lactamase producing enterobacteria including pandemic zoonotic isolates but also as a vector of MRSA.
